# Characteristics of long term survivors of multiple myeloma after autologous stem cell transplantation: a retrospective analysis from a tertiary care centre in India

**DOI:** 10.1016/j.lansea.2025.100680

**Published:** 2025-10-27

**Authors:** Lalit Kumar, Rajegowda Chethan, Prabhat Singh Malik, Raja Pramanik, Ranjit Sahoo, Ahitagni Biswas, Omdutt Sharma, Ritu Gupta, Atul Sharma, Saumya Ranjan Mallick

**Affiliations:** a Department of Medical Oncology, All India Institute of Medical Sciences, New Delhi, 110029, India; b Department of Radiation Oncology, All India Institute of Medical Sciences, New Delhi, 110029, India; c Department of Lab Oncology, All India Institute of Medical Sciences, New Delhi, 110029, India; d Department of Pathology, All India Institute of Medical Sciences, New Delhi, 110029, India

**Keywords:** Very good partial response, Immune signatures, Comorbidities, Causes of death in myeloma, Second primary malignancy

## Abstract

**Background:**

Outcome of newly diagnosed, transplant eligible, multiple myeloma (MM) patients have improved significantly during the past two decades. A small proportion of patients are long term survivors beyond 10 years. We evaluated clinical and laboratory characteristics of these long term survivors.

**Methods:**

Four hundred and thirty eight consecutive MM patients underwent autologous stem cell transplantation (ASCT) between 1995 and 2019. Patients median age was 52 years, (range, 20–73) and 291 (66.4%) were males. 35.3% had ISS stage III disease. 96 (21.9%) patients were long term survivors (LTS), defined as those alive 120 months or more.

**Findings:**

Compared to others, LTS patients more frequently had ISS stage I (42% vs 28.5%, p < 0.001), serum albumin ≥3.5 g/dl (72% vs 57%, p < 0.005), platelet count ≥150,000/μL (88% vs 71%, p < 0.001), eGFR ≥40 ml/min (85.4% vs 75%, p < 0.01), and bone marrow plasma cells ≤40% (60% vs 49%, p < 0.03). Transplant within 12 months of diagnosis (73% vs 51.5%, p < 0.001) and in first remission (89.6% vs 71.3%, p < 0.001) were more common in LTS. Post-ASCT response was superior: CR (80% vs 65.5%, p < 0.004), CR + VGPR (94.8% vs 82.5%, p < 0.001). At median follow-up of 115 months, median overall survival was 264 months and progression-free survival 158 months.

**Interpretation:**

This study identifies a distinct subgroup of MM patients with long-term survival, characterized by favourable baseline features, early and effective treatment, and deep post-transplant responses, with a median progression-free survival exceeding 13 years.

**Funding:**

No funding was received for present study.

**Research in context**

**Evidence before this study**

We searched PubMed to identify studies which have reported on long term survivors with myeloma. Few studies have reported data, mainly from transplant registry or single centre, originating in North America and Western Europe. None of these is from South East Asia/resource limited settings.

**Added value of this study**

This study provides current and comprehensive description of clinical and laboratory Characteristics in relatively a large cohort of MM patients treated uniformly at a single centre in North India, Data was maintained prospectively. A long median follow up of 115 months have allowed us to evaluate impact of transplant outcome plus life style related conditions and second malignancies, in addition to primary malignancy as a cause of death.

**Implications of all the available evidence**

Overall results indicate that patients with low volume/low risk disease treated with novel agents based induction followed by consolidation with autologous stem cell transplantation and long term maintenance are long term survivors. Management of life style related conditions/co-morbidities and prevention of second malignancy are likely to be key areas for future research to reduce morbidity and mortality.

## Introduction

Introduction of novel agents based induction, autologous stem cell transplantation (ASCT), and use of maintenance therapy and better understanding of biology has led to improved survival for newly diagnosed multiple myeloma (NDMM). Currently, induction with bortezomib, lenalidomide and dexamethasone (triplet) with or without daratumumab (quadruplet) is the standard approach.^1^, ^2^, ^3^, ^4^ This is followed by consolidation with ASCT in eligible patients An estimated median overall survival (OS) for transplant recipient is 8.5–10 years in most studies; approximately 15–20% are estimated to be alive beyond 15 years. A few studies have reported actual long term outcome beyond 10 years.^5^, ^6^, ^7^, ^8^, ^9^, ^10^, ^11^, ^12^ We hypothesized that these long term survivors (LTS, ≥120 months) may have distinct clinical and laboratory features. Identification of these characteristics may help to predict the post-transplant outcome and personalize schedule and duration of maintenance therapy. We have analysed the data of 438 consecutive MM patients who underwent ASCT till December, 2019 at our centre; 96 (21.9%) had survival ≥120 months. Evaluation of this ‘LTS Group’ was primary endpoint. Response to transplant, comparison with early relapse (OS ≤ 24 month) and those with OS >24 months to < 120 months (Intermediate Group) were secondary endpoints. We also analysed predictive factors for progression free (PFS) and OS. This report describes the results.

## Methods

Between 1995 and 2019, 459 MM patients underwent high dose melphalan followed by ASCT at our centre; 438 engrafted and are the subjects of this report. 55 (12.6%) patients had survival ≤24 months from date of transplant (Group I, Early Relapse). 96 (21.9%) patients are alive at ≥ 120 months (LTS, Group II) and the remaining 287 (65.5%) patients are alive, >24 months to <120 months (Intermediate, Group III). Clinical, laboratory and transplant related characteristics of these three groups are shown in.Table 1

**Table 1:** Patients characteristics.

Variable	Total no of Pts N (%)	OS ≤ 24 months N = 55 (Group I)	OS ≥ 120 months N = 96, LTS (Group II)	OS > 24–<120 months N = 287 (Group III)	p value
Age (Years)					
Median (Range)	52 (20–72)	54 (29–65)	51.5 (29–67)	52 (20–72)	0.253
≤52	221 (50.5)	22 (40.0)	52 (54.2)	147 (51.2)	
>52	217 (49.5)	33 (60.0)	44 (45.8)	140 (48.8)	
≤60	359 (82.0)	44 (80.0)	80 (83.3)	235 (81.9)	0.918
>60	79 (18.0)	11 (20.0)	16 (16.7)	52 (18.1)	
Gender					
Male	291 (66.4)	33 (60.0)	65 (67.7)	193 (67.2)	0.526
Female	147 (33.6)	22 (40.0)	31 (32.3)	94 (32.8)	
ISS Stage (n = 436)					
I	136 (31.2)	10 (18.2)	39 (40.6)	87 (30.5)	0.001
II	146 (33.5)	13 (23.6)	40 (41.7)	93 (32.6)	
III	154 (35.3)	32 (58.2)	17 (17.7)	105 (36.8)	
M Protein isotype (n = 433)					
IgG	258 (59.6)	38 (70.4)	63 (65.6)	157 (55.5)	0.108
IgA	72 (16.6)	08 (14.8)	14 (14.6)	50 (17.7)	
K + L	103 (23.8)	08 (14.8)	19 (19.8)	76 (26.9)	
Extramedullary disease, (n = 437)					
Yes	95 (21.7)	24 (43.6)	19 (19.8)	52 (18.2)	0.001
No	342 (78.3)	31 (56.4)	77 (80.2)	234 (81.8)	
Hb (G/dl)					
Median (Range)	9.5 (3.2–16)	8.4 (3.5–16)	9.9 (5.1–15.2)	9.5 (3.2–15.8)	0.229
<10	245 (55.9)	35 (63.6)	49 (51.0)	161 (56.1)	
≥10	193 (44.1)	20 (36.4)	47 (49.0)	126 (43.9)	
Platelets (×10^9^/L) N = 415 25–824 × 10^9^					
Median (Range)	195 × 10^9^/L (25–824)	175 × 10^9^/L (48–444)	219 × 10^9^/L (42–617)	190 × 10^9^/L (85–824)	0.001
<150	105 (25.3)	21 (44.7)	11 (12.0)	73 (26.4)	
≥150	310 (74.7)	26 (55.3)	81 (88.0)	203 (73.6)	
Albumin (G/dl)					
Median (Range)	3.7 (1.01–5.70)	3.3 (1.46–5.6)	3.8 (1.8–5.7)	3.7 (61.01–5.60)	0.001
<3.5	174 (39.7)	33 (60.0)	27 (28.1)	114 (39.7)	
≥3.5	264 (60.3)	22 (40.0)	69 (71.9)	173 (60.3)	
eGFR (ml/mt)					
Median (Range)	70.7 (1.7–181.6)	64.0 (1.7–143.0)	74.4 (3.27–124.9)	71.0 (3.8–181.6)	0.021
<40	100 (22.8)	17 (30.9)	14 (14.6)	69 (24.0)	
≥40	338 (77.2)	38 (69.1)	82 (85.4)	218 (76.0)	
β2M (mcg/L) N = 423					
Median (Range)	3940 (810–32578)	5775.0 (1200–53547)	3390 (1279.9–325,578)	4083 (610–35080)	0.001
<5500	274 (64.8)	22 (41.5)	75 (81.5)	235 (81.9)	
≥5500	149 (35.2)	31 (58.5)	17 (18.5)	52 (18.1)	
Serum LDH N = 333					
Median (Range)	223.0 (51–3000)	217.0 (97–1608)	256.0 (51–3000)	218.0 (66–1194.0)	0.310
≤300 IU	232 (69.7)	22 (66.7)	43 (62.3)	167 (72.3)	
>300 IU	101 (30.3)	31 (33.3)	26 (37.7)	64 (27.7)	
FISH N = 142					
Standard	108 (76.1)	8 (61.5)	16 (76.2)	84 (77.8)	0.432
High risk	34 (23.9)	5 (38.5)	05 (23.8)	24 (22.2)	
Serum calcium N = 415					
Median (Range)	9.3 (4.60–15.80)	9.3 (6.0–13.60)	9.2 (4.6–15.4)	9.4 (5.4–15.8)	0.583
<11.0	364 (87.7)	44 (86.3)	81 (89.0)	239 (87.5)	
≥11.0	51 (12.3)	07 (13.7)	10 (11.0)	34 (12.5)	
BM plasma cells N = 435					
Median (range)	40 (1–100)	50.0 (2–100)	35.0 (1–95)	40 (1–100)	0.117
≤40%	225 (51.7)	24 (43.6)	58 (60.4)	143 (50.4)	
>40%	210 (48.3)	31 (56.4)	38 (39.6)	141 (49.6)	
Induction regimen					
Novel agents	350 (79.9)	36 (65.5)	61 (63.5)	253 (88.2)	0.001
VAD	68 (15.5)	10 (18.2)	30 (31.3)	28 (9.8)	
Alk agents	20 (4.6)	09 (16.4)	5 (5.2)	06 (2.1)	
Induction lines					
One line	303 (69.2)	25 (45.5)	71 (74.0)	207 (72.1)	0.002
>One line	135 (30.8)	30 (54.5)	25 (26.0)	80 (27.9)	
Melphalan dose (mg/m^2^)					
≤150	64 (14.6)	10 (18.2)	17 (17.7)	37 (12.9)	0.451
>150	374 (85.4)	45 (81.8)	79 (82.8)	250 (87.1)	
Pre-Tx status					
Sensitive	377 (86.1)	39 (70.9)	85 (88.5)	253 (88.2)	0.002
Resistant	61 (13.9)	16 (29.1)	11 (11.5)	34 (11.8)	
Interval (months) Diagnosis to Tx					
Median (range)	11.5 (2–90.5)	13.5 (4–73)	9.0 (4–90.5)	12.0 (2–81.0)	0.001
0–12	246 (56.2)	24 (43.6)	70 (72.9)	152 (53.0)	
>12	192 (43.8)	31 (56.4)	26 ((27.1)	135 (47.0)	
Transplant in 1st remission					
Primary	330 (75.3)	28 (50.9)	86 (89.6)	215 (74.9)	0.001
Post salvage	108 (26.7)	27 (49.1)	10 (10.4)	72 (25.1)	
Post Tx, day + 100 response					
CR	301 (68.7)	15 (27.3)	77 (80.2)	209 (72.8)	0.001
Others	137 (31.3)	40 (72.7)	19 (19.8)	78 (27.2)	
CR + VGPR	373 (85.2)	28 (50.9)	91 (94.8)	254 (88.5)	0.001
others	65 (14.8)	27 (49.1)	5 (5.2)	33 (11.4)	
CR + VGPR + PR Vs	422 (96.3)	44 (80.0)	96.0 (100.0)	282 (98.3)	0.001
others	16 (3.7)	11 (20.0)	0	05 (1.7)	
CD34+ cells (×10 (6)/kg), N = 412					
<2	73 (17.7)	6 (12.8)	30 (34.5)	37 (13.3)	0.001
≥2	339 (82.3)	41 (87.2)	57 (65.5)	241 (86.7)	
HCT CI score N = 436					
0	160 (36.7)	23 (41.8)	42 (44.7)	95 (33.1)	0.091
≥1	276 (63.3)	32 (58.2)	52 (55.3)	192 (66.9)	
Co-morbidities					
No	124 (28.3)	19 (34.5)	29 (30.2)	76 (26.5)	0.269
Yes	314 (71.7)	36 (65.5)	67 (69.8)	211 (73.5)	
One	159 (50.6%)	18 (11.3%)	35 (22.0%)	106 (66.7%)	
>One	155 (49.4%)	18 (11.6%)	32 (20.6%)	105 (67.7%)	0.222
Diabetes mellitus					
No	368 (84.0)	44 (80.0)	88 (91.7)	236 (82.2)	0.063
Yes	70 (16.0)	11 (20.0)	8 (8.3)	51 (17.8)	
Year of Tx					
1995–2005	76 (17.4)	16 (29.1)	32 (33.3)	28 (9.8)	0.001
2006–15	220 (50.2)	27 (49.1)	64 (66.7)	129 (44.9)	
2016–19	142 (32.4)	12 (21.8)	0	130 (45.3)	
Maintenance N = 438					
Thal	148 (33.8)	12 (21.8)	53 (55.2)	83 (28.9)	0.001
Len	162 (37.0)	13 (23.6)	9 (9.4)	140 (48.8)	
Borte	51 (11.6)	02 (3.6)	11 (11.5)	38 (13.2)	
IFN-a	28 (6.4)	02 (3.6)	17 (17.7)	9 (3.1)	
No	49 (11.2)	26 (47.3)	6 (6.3)	17 (5.9)	
Yes	389 (88.8)	29 (52.7)	90 (93.8)	270 (94.1)	0.001
No	49 (11.2)	26 (47.3)	6 (6.3)	17 (5.9)	

Abbreviations: Thal, thalidomide; Len, lenalidomide; Borte, bortezomib; IFN-a, interferon alfa; Tx, transplant; CR, complete response; VGPR, very good partial response; HCT-CI, hematopoietic cell transplantation comorbidity Index; LTS, Long term survivor; B2M, Beta2microglobulin; VAD, vincristine, adriamycin and dexamethasone.

### Transplant protocol

Briefly, all patients underwent transplant in a single isolation room with reverse barrier nursing. Stem cells were mobilized using G-CSF 10 mcg/Kg in 2 divided doses for 5 days followed by stem cell harvest.^13^ Forty three (9.8%) patients had received plerixafor, if on day 4 peripheral blood CD34 count was <20/μL.^14^ Following stem cell harvest patients received high dose melphalan 200 mg/m^2^, those with renal impairment (eGFR <40 ml/min) received melphalan dose of 140–150 mg/m^2^. Response to transplant was evaluated on day +100 ± one week^15^^,^^16^ and patients were advised maintenance therapy as per protocol.

### Statistics

Base line patients characteristics were compared using Pearson Chi-square test. The prognostic factors for response to transplant were analysed by Pearson Chi-square test and binary logistic regression analysis. The Kaplan and Meier method was used to estimate survival and these were compared using log-rank test. In addition to categorical comparisons, we performed univariable and multivariable Cox proportional hazards analyses using continuous variables for quantitative variables e.g., age, albumin, platelet count etc (Supplementary Table 5A and B). Overall survival (OS) was defined as the time from date of transplant until death or date of censor. Progression free survival (PFS) was calculated from date of transplant to disease progression or death (regardless of the cause of death). The prognostic factors for survival were analysed by Cox regression analysis. Analysis was carried out using SPSS-20 statistical software (IBM, Atlanta, USA). The study was conducted in accordance with the STROBE (Strengthening the Reporting of Observational Studies in Epidemiology) guidelines.^17^ The median follow up for all patients from date of transplant is 115.0 months (95% CI 109.5–120.5). Data has been censored on 30th June, 2024.

### Ethics statement

The study was approved by the Ethics Committee of AIIMS and was conducted in accordance with the principles of the Declaration of Helsinki. In view of retrospective nature of study participants informed consent was waived off.

### Role of the funding source

No funding was received for present work.

## Results

### Patients characteristics

Patients median age was 52 years (range, 20–73 Years), 291 (66.4%) were males and 35.3% patients had ISS stage III disease (Table 1). 103 (23.8%) patients had light chain myeloma, 21.7% had extramedullary disease (EMD) at presentation and 22.8% of patients had eGFR <40 ml/min 23.9% of patients had high risk cytogenetic abnormalities (CyA), 30.3% had elevated serum LDH (>300 IU) and 25.3% of patients had platelets counts <150,000/μL. Median interval from diagnosis to transplant was 11.5 months (range, 2–90.5 months).

Three hundred and fifty patients (79.9%) had received novel agents based induction followed by VAD (Vincristine, Adriamycin, and dexamethasone) as continuous infusion in 68 (15.5%) and alkylating agents based induction in 20 patients (4.6%) before year 2001.

30.7% of patients had received more than one line of induction therapy. Prior to transplant- 377 (86.1%) patients had chemo-sensitive disease (defined as achievement of complete (CR), very good partial response (VGPR) and partial response (PR) as per IMWG criteria.^16^

### Co-morbidities

314 (71.7%) patients had co-morbidities; Of these 159 (50.6%) had one, and 105 (33.4%) -two, 41 (13.1%) three and 9 (2.9%) had four co-morbidities. Most common ones were-obesity (53%),^18^ hypertension (32.4%), diabetes mellitus (16.0%), hypothyroidism (9.8%), chronic obstructive pulmonary disease (5.3%) and coronary artery disease (1.4%), etc. These were almost similarly distributed in the three groups. Frequency of diabetes mellitus was lower in the LTS group (8.3%) compared to Intermediate group (17.8%) and early relapse group (20.0%), p < 0.063 (Table 1).

#### LTS vs others

Comparison of LTS Group (OS ≥ 120 months, n = 96) with remaining patients (n = 342) confirms that patients in LTS group had better clinical and laboratory characteristics—lower proportion of ISS stage III (17.7% vs 40.3%), Platelets <150,000/μL (12.0% vs 29.1%), serum albumin (<3.5 G/dl, 28.1% vs 43.0%), eGFR<40 ml/min (14.6% vs 25.1%), HCT-CI score, ≥1 (55.3% vs 65.5%, short interval from diagnosis to transplant (≤12 months; 73% vs 51.5%), transplant in first remission vs post salvage therapy (89.6% vs 71.3%), and higher response rates to transplant; CR (80.2% vs 65.5%), CR + VGPR (94.8% vs 82.5%) (Table 1).

#### LTS vs early relapse (Group I)

Fifty five patients (12.6%) relapsed within 24 months of transplant and had died **(**Supplementary Table S1). These patients had higher disease burden and high risk disease^19^ as evidenced by high risk cytogenetic abnormalities in 38.5%, extramedullary disease–43.6%, stage ISS III–58.2%, and renal impairment (eGFR <40 ml/min) in 30.9% patients. Other features were–lower platelet counts <150,000/μL in 44.7%, serum albumin <3.5 G/dl in 60.0%, higher serum β2 micro-globulin (≥5500 mcg/L (58.5%), and higher HCT-CI score ≥1 in 58.2% of patients (Supplementary Table S1). More patients in early relapse group had pre transplant chemo-resistant disease (29.1%), required more than one line of induction therapy (54.5%) and had longer interval (>12 months in 56.4%) from diagnosis to transplant. As a result - transplant response rates - CR (p < 0.001), CR + VGPR (p < 0.001) or CR + VGPR + PR (<0.001) were lower. Further, only 52.7% of patients received post-transplant maintenance therapy compared to 94% in LTS group, p < 0.001. Age, gender, myeloma isotype and melphalan dose (140 vs 200 mg/m^2^) were not different in the two groups (Table 1 and Supplementary Table S1). Clinical and laboratory characteristics of LTS Group vs remaining patients (n = 342) are given in Supplementary Table S2. Patients in LTS Group had low risk disease compared to early relapse and Intermediate Group combined (Supplementary Table S2).

### Post transplant maintenance therapy

Post transplant 389 patients (88.8%) had received maintenance therapy; low dose thalidomide (50 mg/day) (33.8%), lenalidomide (10 mg/day × 21 days then one week off) (37%), bortezomib day 1 and 15 (11.6%) and interferon alfa (6.4%) as per prevalent protocols in different periods. 49 patients (11.2%) patients refused or did not receive maintenance therapy. These were advised close observation with supportive care including zoledronic acid, calcium and vitamin D supplements. 52.7% patients in Group I did not receive maintenance therapy compared to 6.5% in LTS and 5.9% in the Intermediate group (Group III) (Table 1).

### Response to transplant (Table 2)

Overall 373 (85.2%) patients had significant response; CR-301 (68.7%), VGPR-72 (16.4%). Response rates were superior in LTS Group for each response category; CR (80.2% vs 65.5% p < 0.004), CR + VGPR (94.8% vs 82.5%, p < 0.001) and CR + VGPR + PR (100.0% vs 95.4%, p < 0.01), compared to others (Table 2).

**Table 2:** Response to transplant.

Response	Total no of Pts (%) N = 438	Early relapse OS (≤24 months), N = 55 Group I		LTS (OS ≥ 120 months) N = 96 Group II		P Value Group I vs II	Intermediate OS < 120 months N = 287 Group III		Group II vs III p value
		N	%	N	%		N	%	
Complete	301 (68.7)	15	27.3	77	80.2		209	72.8	
VGPR	72 (16.4)	13	23.6	14	14.6		45	15.7	
CR + VGPR	373 (85.2)	28	50.9	91	94.8	0.002	254	88.5	0.063
Partial (PR)	49 (11.2)	16	29.1	5	5.2		28	9.8	
CR + VGPR + PR	422 (96.3)	44	80.0	96	100.0	0.001	282	98.3	0.247
Stable	13 (3.0)	8	14.5	0	–		5	1.7	
Not evaluable	03 (0.7)	3	5.5	0	–		–	–	
Total	438 (100.0)	55		96			287		

CR-complete response, VGPR-very good partial response, PR-partial response, LTS-m long term survival. Response Rates for LTS versus others (n = 342): CR 80.2% vs 65.5%, p < 0.004, CR + VGPR (94.8% vs 82.5%, p < 0.001, CR + VGPR + PR (100% vs 95.4%, p < 0.018).

### Measurable residual disease (MRD)

MRD assessment using Flow cytometry and PET scan was available for 118 patients transplanted between April, 2016 and November, 2019.^15^ 84 (71.2%) patients were MRD negative and 34 (28.8%) were MRD positive. Most of these patients belonged to intermediate group (Group III (OS > 24 months–<120 months).

### Overall survival

**Fig. 1:**
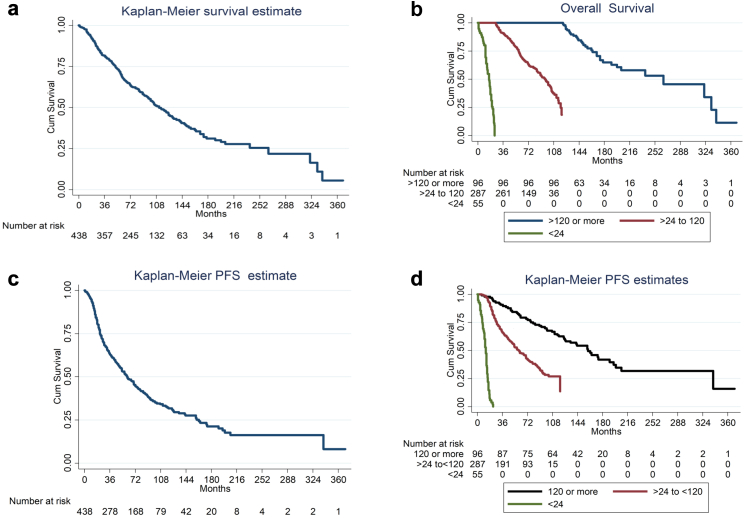
a: Overall Survival. b: Overall Survival in three Groups. c: Progression free Survival. d: Progression free Survival according to Groups.

Estimated median OS is 112.0 months (95% CI 96.3–127.7) for all patients; being superior for LTS (Group II)—264 months (95% CI 171.2–356.8) compared to 16 months (95% CI 12.8–19.2) for early relapse (Group I), and 96 months (95% CI 88.4–103.6) for the intermediate group (Group III), p < 0.001 and 82 months (95% CI 70.1–93.9) for Group I and III combined, p < 0.001 (Fig. 1A and B). For all patients–10 Years OS probability is 47.7% ± 0.03(SE).

Median OS is superior for each response category (CR, CR + VGPR and CR + VGPR + PR) in the LTS Group compared to others (Table 3). On multivariable analysis—in the LTS group—presence of pretransplant chemosensitive disease and achievement of CR post transplant were important predictors. In the remaining patients (n = 342)–absence of extramedullary disease, albumin (≥3.5 G/dl), year of transplant, achievement of CR to transplant, and use of maintenance therapy were important predictive factors. (Supplementary Table S3. For quantitative laboratory parameters, we analysed both for categorical as well as continuous variables. Continuous modelling helped to detect effects of Hb, eGFR, BM plasma cells), while categorical analysis highlighted clinically relevant thresholds (e.g., β2-microglobulin, platelet count). Importantly, albumin emerged as a consistent predictor across both approaches. We believe this complementary strategy strengthens the interpretability and may be considered for analysis (Supplementary Table S3A).

**Table 3:** Survival according to transplant response.

	Total N = 438	Group I N = 55 (OS ≤ 24 months)	LTS (Group II) N = 96 (OS ≥ 120 months)	Group III N = 287 (OS > 24–<120 months)	p value
**Median overall survival in months**					
**Median OS (95% CI)**	**112.0 (96.3–127.7)**	**16.0 (12.8–19.2)**	**264.0 (171.2–356.8)**	**96 (88.4–103.6)**	**0.001**
CR N = 301	156.0 (125.0–187.0)	N = 15	N = 77	N = 209	0.001
		17.0 (10.8–23.2)	322.0 (177.5–466.5)	107.0 (96.4–117.6)	
VGPR N = 72	58.0 (38.6–77.4)	N = 13	N = 14	N = 45	0.001
		19.0 (13.3–24,7)	238.0 (−)	58.0 (39.6–76.4)	
CR + VGPR N = 373	131.0 (106.7–155.3)	N = 28	N = 91	N = 254	0.001
		17.0 (12.9–21.1)	264.0 (181.9–346.1)	100.0 (93.1–106.9)	
PR = 49	51.0 (29.1–72.9)	N = 16	N = 5	N = 28	0.001
		18.0 (13.1–22.9)	141.5 (129.3–153.8)	59.0 (48.0–70.0)	
CR + VGPR + PR N = 422	119.0 (103.5–134.5)	N = 44	N = 96	N = 282	0.001
		17.5 (15.9–19.1)	264.0 (171.2–356.8)	97.0 (88.7–105.3)	

		Group I N = 55	Group II N = 96	Group III N = 287	p value
**Median progression free survival in months**					
**Median PFS (95% CI)**	**60.0 (50.8**–**69.2)**	**12.0 (10.3**–**13.7)**	**158.0 (125.3**–**190.7)**	**60.0 (50.7**–**69.3)**	**0.001**
CR	91.0 (74,1–107.9)	11.0 (8.5–13.5)	164.0 (147.3–180.7)	76.0 (63.9–88.1)	0.001
VGPR	64.8 (18.8–25.2)	14.0 (12.3–15.7)	70.0 (32.4–107.6)	22.0 (18.2–25.8)	0.001
CR + VGPR	62.0 (50.8–69.2)	13.0 (10.9–15.1)	162.0 (131.5–192.5)	66.0 (56.4–75.6)	0.001
PR	18.0 (14.0–22.0)	13.0 (11.04–15.0)	18.0 (0.82–35.2)	28.0 (21.5–34.5)	0.001
CR + VGPR + PR	60.0 (50.8–69.2)	13.0 (11.4–14.6)	158.0 (129.4–186.6)	59.0 (49.6–68.4)	0.001

Group I and III: Median OS: 81.0 (69.7–92.3), Median PFS: 45.0(36.5–53.5).

Figures in bracket represent 95% CI, CR, complete response; VGPR, very good partial response; PR, partial response.

### Progression free survival

Median PFS was 60 months (95% CI 50.8–69.2) for all patients; being superior for LTS Group, 158 months (95% CI 125.3–190.7) compared to 12.0 (95% CI 10.3–13.7) in the early relapse group (Group I) (p < 0.001), and 60 months (95% CI 50.7–69.3) in the Intermediate group (Group III) and 45.0 months (95% CI 36.5–53.5) for Group I and III, combined, p < 0.001 (Fig. 1c and d). For all patients–10 Years PFS probability is 27.6% ± 0.05(SE). Median PFS was superior for each response category in the LTS Group compared to others (Table 5).

**Table 5:** Progression free survival: univariate analysis.

Variable	LTS (Group II) OS ≥ 120 months N = 96	Median PFS (Mon)	95% CI	p value	Others (Group I + III) N = 342	Median PFS (Mon),	95% CI	p value
Age (Years)								
≤52	52 (50.5)	164.0	117.4–210.6	0.809	169 (49.4)	46.0	54.3–57.7	0.555
>52	44 (45.8)	158.0	117.3–198.7		173 (50.6)	44.5	33.3–55.7	
≤60	80 (83.3)	164.0	135.3–192.7	0.051	279 (81.6)	39.5	30.5–48.5	0.124
>60	16 (16.7)	115.0	36.3–193.7		63 (18.4)	61.0	42.9–79.1	
Gender								
Male	65 (67.7)	158.0	125.8–190.2	0.499	226 (66.1)	44.5	35.7–53.3	0.183
Female	31 (32.3)	174.0	99.5–248.5		116 (33.9)	49.0	21.9–76.1	
ISS stage (n = 436)								
I	39 (40.6)	142.0	105.9–178.1	0.612	97 (28.5)	42	12.7–71.3	0.181
II	40 (41.7)	164.0	123.3–204.8		106 (31.2)	51.0	42.2–59.8	
III	17 (17.7)	173.0	44.4–301.6		137 (40.3)	37.0	24.2–49.8	
M Protein isotype (n = 433)								
IgG	63 (65.6)	158.0	119.0–197.0	0.325	195 (57.9)	52.5	40.0–65.0	0.970
IgA	14 (14.6)	134.0	37.5–230.5		58 (17.2)	42.0	26.5–57.5	
K + L	19 (19.8)	–	–		84 (24.9)	42.0	24.5–59.5	
EM disease, (n = 437)								
Yes	19 (19.8)	97.0	47.2–146.8	0.076	76 (22.3)	24.0	17.4–30.6	0.091
No	77 (80.2)	164.0	146.9–181.1		265 (77.7)	51.0	43.4–58.6	
Hb (G/dl)								
<10	49 (51.0)	158.0	109.2–206.8	0.679	196 (57.3)	43.0	32.4–53.6	0.331
≥10	47 (49.0)	159.0	115.1–202.9		146 (42.7)	52.5	40.2–64.8	
Platelets (×10^9^/L) N = 415								
<150	11 (12.0)	–	–	0.706	94 (29.1)	35.0	18.8–51.2	0.477
≥150	81 (88.0)	158.0	130.3–185.7		229 (70.9)	52.5	41.6–63.4	
Albumin (G/dl)								
<3.5	27 (28.1)	158.0	47.2–268.8	0.288	147 (43.0)	42.5	25.1–59.9	0.064
≥3.5	69 (71.9)	162.0	129.6–194.4		195 (57.0)	46.5	32.4–60.6	
eGFR (ml/mt)								
<40	14 (14.6)	111.0	–	0.909	86 (25.1)	42.5	26.1–58.9	0.686
≥40	82 (85.4)	159.0	129.4–188.6		256 (74.9)	48.5	37.1–59.9	
β2M (mcg/L) N = 423								
<5500	75 (81.5)	158.0	127.7–188.3	0.530	199 (59.8)	51.0	41.3–60.7	0.073
≥5500	17 (18.5)	173.0	44.4–301.6		134 (40.2)	35.0	21.8–48.2	
Serum LDH N = 333 U/L								
≤300	43 (62.3)	196.0	157.2–234.8	0.940	189 (71.6)	57.0	44.0–70.0	0.016
>300	26 (37.37)	190.0	104.9–275.1		75 (28.4)	34.5	18.5–50.5	
FISH N = 142								
Standard	16 (76.2)	111.0	77.7–144.3	0.362	92 (76.0)	84.0	67.4–100.6	0.001
High risk	05 (23.8)	142.0	45.4–238.6		29 (24.0)	56.5	32,3–80.7	
S. Calcium N = 415								
<11.0	81 (89.0)	158.0	130.6–185.4	0.352	283 (87.3)	44.0	34.7–53.3	0.797
≥11.0	10 (11.0)	173.0	128.4–217.6		41 (12.7)	44.5	15.6–73.4	
BM Plasma cells, N = 435								
≤40%	58 (60.4)	143.0	108.7–177.3	0.157	167 (49.3)	53.0	37.1–68.9	0.127
>40%	38 (39.6)	190.0	158.2–221.8		172 (50.7)	38.0	27.3–48.7	
Induction regimen								
Novel	61 (63.5)	–	–	0.081	289 (84.5)	57.0	46.5–67.5	0.001
VAD	30 (31.3)	142.0	99.1–184.9		38 (11.1)	18.0	15.0–21.0	
Alkylat	5 (5.2)	107.0	158.2–221.8		15 (4.4)	18.0	10.4–25.6	
Induction lines (n = 437)								
One	71 (74.0)	164.0	133.9–194.1	0.124	232 (67.8)	61.0	50.6–71.4	0.001
>One	25 (26.0)	127.0	69.1–184.9		110 (32.2)	23.0	19.4–24.6	
Melphalan dose (mg/m^2^)								
>150	17 (17.7)	126.0	83.2–168.8	0.698	47 (13.7)	38.0	16.5–59.5	0.827
≤150	79 (82.3)	162.0	136.6–187.4		295 (86.3)	46.5	37.8–55.2	
Pre-Tx status								
Sensitive	85 (88.5)	164.0	146.6–181.4	0.004	292 (85.4)	53.0	44.9–61.1	0.001
Resistant	11 (11.5)	77.0	0–156.8		50 (14.6)	20.0	15.4–24.6	
Interval diagnosis to Tx (months)								
0–12	70 (72.9)	173.0	129.5–216.5	0.302	176 (51.5)	49.0	38.5–59.5	0.263
>12	26 ((27.1)	143.0	103.3–182.7		166 (48.5)	38.0	24.4–51.6	
Transplant in 1st remission								
Primary	86 (89.6)	162.0	134.7–189.3	0.345	244 (71.3)	76.0	63.2–88.8	0.001
Post salvage	10 (10.4)	127.0	81.8–172.2		98 (28.7)	24.0	20.1–27.9	
Post Tx, d + 100 response								
CR	77 (80.2)	164.0	147.3–180.7	0.001	224 (65.5)	70.5	59.1–81.9	0.001
Others	19 (19.8)	77.0	28.8–111.2		118 (34.5)	18.0	15.5–20.5	
CR + VGPR	91 (94.8)	162.0	135.5–188.5	0.001	282 (82.5)	56.5	46.7–66.3	0.001
Others	5 (5.2)	18.0	0.823–35.2		60 (17.5)	16.0	13.9–18.1	
CD34 + cells (×10 (6)/kg) N = 412								
<2	30 (34.5)	158.0	132.2–183.8	0.946	43 (13.2)	30.0	19.9–40.1	0.317
≥2	57 (65.5)	174.0	119.6–228.4		282 (86.8)	53.0	44.4–61.6	
HCT CI score N = 436								
0	42 (44.7)	162.0	109.8–214.2	0.685	118 (34.5)	39.0	26.3–51.7	0.574
≥1	52 (55.5)	158.0	82.8–233.2		224 (65.5)	47.0	37.2–56.8	
Year of transplant								
<2005	32 (33.3)	158.0	116.4–199.6	0.588	44 (12.9)	18.0	14.8–21.2	0.001
2006–15	64 (66.7)	162.0	111.0–213.0		156 (45.6)	32.0	24.4–39.6	
2016–19	0	–			142 (41.5)	–	–	
Co-morbidities								
No	29 (30.2)	158.0	123.3–192.7	0.594	94 (27.5)	69.5	45.7–93.3	0.894
Yes	67 (69.8)	162.0	118.7–205.3		248 (72.5)	60.0	51.0–69.0	
Diabetes Mellitus								
No	88 (91.7)	158.0	130.3–185.7	0.230	280 (81.9)	45.0	36.2–53.8	0.379
Yes	8 (8.3)	339.0	–		62 (18.1)	38.0	12.0–64.0	
Maintenance N = 438								
Yes	90 (93.8)	159.0	131.2–186.8	0.792	299 (87.4)	56.5	46.6–66.4	0.001
No	6 (6.3)	127.0	–		43 (12.6)	13.5	9.6–17.4	

Abbreviations: PFS, progression free survival; mon, months; CR, complete response; VGPR, very good partial response; EM, Extra-medullary; BM, bone marrow; HCT-CI, Hematopoietic cell transplantation co-morbidity index.

On multivariable analysis–achievement of post-transplant (day +100)—CR, CR + VGPR, absence of extramedullary disease, and age <60 years were predictive of PFS for LTS Group. For the remaining patients (n = 342)—presence of pre-transplant chemo-sensitive disease, high risk cytogenetics, absence of extramedullary disease, serum albumin (≥3.5 G/dl), achievement of CR (on day +100) and use of maintenance therapy were important predictors (Supplementary Table S5A.

### Current status

In the LTS Group-66.7% of patients are alive (disease free-43.8%, in CR2-12.5%, with disease-7.3%, biochemical relapse-1.0%, and with MGUS like -2.1%). A total of 33.3% of patient have died, primarily due to disease-26.0% and 7.3% due to unrelated causes). In the remaining patients (Group I and III) (n = 342)- 41.3% patients are alive and 58.7% have died (Supplementary Table S6).

## Discussion

In present study we analysed clinical and laboratory characteristics of MM patients who underwent ASCT and were alive beyond 10 years (LTS Group). These results were compared with remaining patients including early relapse (OS ≤ 24 months) and intermediate group (OS 24–<120 months (Table 1 and Supplementary Table S1 and S2). Pre-transplant chemo-sensitive disease, achievement of CR or CR + VGPR post-transplant (day +100) and use of maintenance therapy were predictors of improved PFS and OS for LTS. We acknowledge that some patients classified in the intermediate survival group may ultimately survive beyond 10 years. Their current classification reflects minimum observed survival, not final outcome.

In general, patients in LTS group had low disease burden. In contrast, early relapse group patients had high risk disease and higher disease burden^19^ as evidenced by higher proportion of patients with ISS III, extramedullary disease, high risk CyA, renal impairment, platelet counts <150,000/μL, low serum albumin (<3.5 G/dl. More patients underwent transplant in second remission (post salvage therapy), had received > one line of induction regimen, and had longer interval from diagnosis to transplant (>12 months). These differences led to lower transplant response rates (CR + VGPR) and coupled with lack of use of maintenance therapy contributed to poor outcome (Table 1, Supplementary Table S1). These observations are in line with earlier and contemporary studies suggesting that pre-transplant disease characteristics are determinant of posttransplant response^5^, ^6^, ^7^, ^8^, ^9^, ^10^, ^11^, ^12^, ^13^^,^^20^ (Supplementary Table S7). Identification of such high risk patients, in time, calls for alternative strategies-in form of aggressive induction, early consolidation with single or double transplant and dual maintenance therapy with or without daratumumab.^21^, ^22^, ^23^, ^24^

Stage of myeloma (ISS)–is an important predictor of outcome. Overall, patients with ISS III had inferior PFS and OS (Supplementary Table S1). Outcome was not different among 3 stages in the LTS Group, possibly due to small no (Table 4). Similar to earlier observations-patients who were transplanted in second remission after salvage therapy (26.7%) and those who had received more than one line of induction therapy (30.7%) had inferior outcome.^25^ While high risk cytogenetics was predictor of inferior outcome in Group I and III, no of patients with high risk cytogenetics were small in LTS Group (Table 4).

**Table 4:** Overall survival: univariate analysis.

Variable	LTS (Group II) OS ≥ 120 months N = 96	Median OS (Mon)	95% CI	p value	Others (Group I + III) N = 342	Median OS (Mon),	95% CI	p value
Age (Years)								
≤52	52 (54.2)	264.0	210.1–317.9	0.158	169 (49.4)	83.5	60.1–102.9	0.269
>52	44 (45.8)	198.0	89.3–306.7		173 (50.6)	81.0	68.7–93.9	
≤60	80 (83.3)	264.0	213.0–315.0	0.027	279 (81.6)	74.0	60.7–85.3	
>60	16 (16.7)	156.0	130.9–181.1		63 (18.4)	85.0	77.1–116.9	0.603
Gender								
Male	65 (67.7)	238.0	146.6–329.4	0.559	226 (66.1)	75.0	61.01–85.3	0.001
Female	31 (32.3)	264.0	141.5–386.5		116 (33.9)	89.0	77.1–116.9	
ISS stage (n = 436)								
I	39 (40.6)	238.0	92.6–383.4	0.686	97 (28.5)	100.0	71.2–128.8	0.033
II	40 (41.7)	264.0	187.8–340.2		106 (31.2)	85.0	73.3–97.8	
III	17 (17.7)	–			137 (40.3)	63.0	53.1–72.9	
M Protein isotype (n = 433)								
IgG	63 (65.6)	238.0	178.6–297.4	0.379	195 (57.9)	84.0	69.6–98.4	0.263
IgA	14 (14.6)	332.0	175.9–488.1		58 (17.2)	70.0	52.3–87.7	
K + L	19 (19.8)	–	–		84 (24.9)	98.5	71.7–125.3	
EM disease, (n = 437)								
Yes	19 (19.8)	–	–	0.696	76 (22.3)	40.0	17.2–62.8	0.001
No	77 (80.2)	264.0	167.4–360.6		265 (77.7)	88.0	78.0–98.0	
Hb (G/dl)								
<10	49 (51.0)	332.0	199.9–464.1	0.322	196 (57.3)	70.0	56.0–84.0	0.140
≥10	47 (49.0)	238.0	153.4–322.6		146 (42.7)	89.0	73.9–104.1	
Platelets (×10^9^/L) N = 415								
<150	11 (12.0)	169.0	153.1–184.9	0.4460	94 (29.1)	85.0	48.0–122.5	0.595
≥150	81 (88.0)	264.0	176.9–351.1		229 (70.9)	83.5	72.0–95.0	
Albumin (G/dl)								
<3.5	27 (28.1)	204.0	185.6–222.4	0.282	147 (43.0)	70.0	52.0–88.0	0.017
≥3.5	69 (71.9)	264.0	175.5–350.5		195 (57.0)	90.5	75.5–105.5	
eGFR (ml/mt)								
<40	14 (14.6)	340.7	–	0.783	86 (25.1)	79.0	51.7–106.3	0.714
≥40	82 (85.4)	274.02	181.0–347.0		256 (74.9)	83.5	71.0–96	
β2M (mcg/L) N = 423								
<5500	75 (81.5)	264.0	185.9–342.1	0.473	199 (59.8)	91.5	76.8–106.2	0.022
≥5500	17 (18.5)	–	–		134 (40.2)	65.0	49.8–80.2	
Serum LDH N = 333 U/L								
≤300	43 (62.3)	238.0	–	0.513132	189 (71.6)	100.0	86.2–113.8	0.032
>300	26 (37.37)	–	–		75 (28.4)	74.0	55.8–92.2	
FISH N = 142								
Standard	16 (76.2)	–	–	0.842	92 (76.0)	105.0	91.0–119.0	0.001
High risk	05 (23.8)	–	–		29 (24.0)	64.0	31.0–97.0	
S. Calcium N = 415								
<11.0	81 (89.0)	322.0	163.2–480.8	0.636	283 (87.3)	83.5	70.5–96.5	0.953
≥11.0	10 (11.0)	264.0	–		41 (12.7)	73.0	12.7–133.3	
BM plasma cells, N = 435								
≤40%	58 (60.4)	238.0	174.8–301.2	0.137	167 (49.3)	89.0	73.1–104.9	0.128
>40%	38 (39.6)	339.0	192.0–486.0		172 (50.7)	67.0	49.3–84.7	
Induction regimen								
Novel	61 (63.5)	–	–	0.198	289 (84.5)	91.5	82.2–100.8	0.001
VAD	30 (31.3)	204.0	134.2–273.8		38 (11.1)	50.5	33.9–67.1	
Alkylat	5 (5.2)	174.0	119.3–228.8		15 (4.4)	23.0	18.0–28.00	
Induction lines (n = 437)								
One	71 (74.0)	264.0	177.5–350.5	0.0695	232 (67.8)	97.0	88.0–106.0	0.001
>One	25 (26.0)	163.0	145.0–181.0		110 (32.2)	47.0	34.9–59.1	
Melphalan dose (mg/m^2^)								
≤150	17 (17.7)	322.0	36.3–607.7	0.9029	47 (13.7)	72.0	50.0–93.5	0.364
>150	79 (82.3)	264.0	185.0–343.0		295 (86.3)	83.5	70.0–97.0	
Pre-Tx status								
Sensitive	85 (88.5)	299.9	188.9–455.1	0.004	292 (85.4)	91.0	81.2–100.8	0.001
Resistant	11 (11.5)	238.0	143.6–182.4		50 (14.6)	40.0	27.3–52.7	
Interval diagnosis to Tx (months)								
0–12	70 (72.9)	264.0	176.5–351.5	0.746	176 (51.5)	89.0	78.8–99.2	0.081
>12	26 (27.1)	204.0	–		166 (48.5)	68.0	54.9–81.1	
Transplant in 1st remission								
Primary	86 (89.6)	264.0	178.7–349.3	0.279	244 (71.3)	96.0	86.8–105.2	0.001
Post salvage	10 (10.4)	174.0	142.4–205.6		98 (28.7)	45.0	32.7–57.3	
Post Tx, d + 100 response								
CR	77 (80.2)	322.0	177.5–466.7	0.035	224 (65.5)	104.0	95.0–113.0	0.001
Others	19 (19.8)	174.0	115.9–232.1		118 (34.5)	35.0	27.0–43.0	
CR + VGPR	91 (94.8)	264.0	181.9–346.6	0.001	282 (82.5)	91.5	83.0–100.0	0.001
others	5 (5.2)	141.5	129.3–153.8		60 (17.5)	28.5	18.4–38.6	
CD34 + cells (×10 (6)/kg)								
<2	30 (34.5)	190.0	126.2–253.8	0.273	43 (13.2)	72.0	45.5–98.5	0.097
≥2	57 (65.5)	264.0	226.7–301.3		282 (86.8)	88.0	73.9–102.1	
HCT CI score N = 433								
0	42 (44.7)	264.0	178.0–350.0	0.436	118 (34.5)	80.6	54.4–108.6	0.908
≥1	52 (55.3)	238.0	144.5–331.5		224 (65.5)	80.3	67.4–90.6	
Year of transplant								
<2005	32 (33.3)	238.0	157.0–319.4	0.560	44 (12.9)	37.0	18.6–55.4	0.001
2006–15	44 (66.7)	–	–		156 (45.6)	63.0	54.0–72.0	
2016–19	0				142 (41.5)	NR	–	
Co-morbidities								
No	29 (30.2)	322.0	150.9–493.1	0.9587	94 (27.5)	85.0	60.6–101.4	0.512
Yes	67 (69.8)	264.0	185.5–342.5		248 (72.5)	82.0	71.8–96.2	
Diabetes mellitus								
No	88 (91.7)	–	–	0.428	280 (81.9)	82.0	67.5–96.4	0.836
Yes	8 (8.3)				62 (18.1)	75.0	50.1–99.9	
Maintenance N = 438								
Yes	90 (93.8)	264.0	170.6–357.4	0.763	299 (87.4)	91.5	83.0–100.0	0.001
No	6 (6.3)	174.0	154.0–194.0		43 (12.6)	19.0	10.0–28.0	

Abbreviations: PFS, progression free survival; mon, months; CR, complete response; VGPR, very good partial response; EM, Extra-medullary; BM, bone marrow; HCT-CI, Hematopoietic cell transplantation co-morbidity index.

Depth of response –(achievement of CR and/or VGPR) is an important predictor of outcome after ASCT. In present study post-transplant achievement of CR and CR + VGPR were strong predictors of long OS overall and PFS on multivariable analysis, similar to earlier studies.^5^, ^6^, ^7^, ^8^, ^9^, ^10^, ^11^, ^12^^,^^20^^,^^26^, ^27^, ^28^, ^29^, ^30^ These results are also similar to studies published from India.^31^^,^^32^ Recent studies have shown that post ASCT -negative measurable (minimal) residual disease (MRD) status is a better predictor (than CR/VGPR) for PFS and OS and should be aimed for.^33^^,^^34^ In present study, MRD data was available (mainly for the intermediate group). This along with PET scan negativity (accounting for extramedullary disease) is possibly a better predictor.^35^ Sustained MRD negativity at 1 year or 3 years may identify a subgroup of patients likely to remain myeloma free.^36^^,^^37^

Considering the fact that myeloma now resembles a chronic illness due to treatment advances-a number of other factors e.g., life style related diseases/comorbidities (e.g. obesity, hypertension, diabetes mellitus, coronary artery disease, chronic lung disease etc)^38^^,^^39^ and development of second primary malignancies (SPM)^40^ may also influence overall survival. These could contribute to premature mortality while myeloma being in remission.^38^, ^39^, ^40^ In present study 11 (2.5%) patients developed second malignancy: acute myeloid leukemia-2, acute lymphoblastic leukemia-2, renal cell carcinoma-2, tongue cancer-2, hepatocellular carcinoma-2, prostate cancer-1); in 6 of them myeloma was in remission at the diagnosis of SPM. Coronary artery disease (n = 7) cerebral haemorrhage (stroke) (n = 1), viral infections (dengue-2, covid-19 in 2, influennza-1), PCP pneumonia-1, Alzheimer's (n = 1), and suicide (n = 1) led to premature death in a few patients (Supplementary Table S6). Thus, in context of prolonged survival-risk of SPM and complications due to life style related diseases, though low at present but these rates are likely to change with time and increasing age and as newer treatment regimens evolve.^38^, ^39^, ^40^

Similar to earlier studies, here too, outcome for myeloma patients have improved with time. This is reflective of improved induction therapy (with novel agents) with higher response rates which translated in better depth of response post-transplant (CR, CR + VGPR), use of maintenance therapy and steady improvement in PFS and OS and reduction in myeloma related deaths (Supplementary Table S8). We did not see a clear plateau in long term survivors underscoring the fact that risk of disease relapse continue to remain even after a long remission. It has been hypothesized that unique immune signatures might contribute to long term remissions in both transplant and non-transplant setting; these include—recovery of polyclonal immunoglobulin at one year post transplant,^41^ more circulating CD8+ T cells and lower CD4/CD8 ratio,^42^ high frequency of cytotoxic T-cell clonal expansions, a lower Treg/Th17 ratio,^43^ a higher proportion of CD4+ and CD8+ effector memory T-cells, a particular redistribution of inhibitory and activating receptors in NK-cells, an increase in naïve B cells and a reduction in marginal zone-like and class-switched memory B-cells.^44^ Availability of cytogenetics data in one third of patients and MRD data mainly for intermediate group are important limitations of the present study. Nonetheless a long term follow up of patients treated uniformly in this real world setting in a single centre study suggested that 22% of patients have achieved long term survival. Post-transplant attainment of CR and VGPR is the most important predictor of long term survival. We could also identify a number of clinical and laboratory variables associated with higher disease burden and inferior outcome.

## Contributors

Lalit Kumar: Concept and design, data review, manuscript writing; Chetan R:Data collection, clinical management; Prabhat S Malik; Clinical Management; Ranjit Kumar Sahoo: Data interpretation, manuscript review, discussion; Raja Pramanik:Clinical Management; Ahitagni Biswas: Clinical Management; Omdutt Sharma:Laboratory data management; Ritu Gupta:Review of Laboratory data; Atul Sharma: Clinical data management; Saumya Ranjan Mallick: Collaborated in data collection, discussion.

The authors had full access to all data and take full responsibility for its integrity and the accuracy of the analysis.

## Data sharing statement

Individual participant data supporting the conclusions of this article will be made available by the authors on request.

## Declaration of interests

All authors declare that there is no conflict of interest.
