# Supplementary material for: Characteristics of long term survivors of multiple myeloma after autologous stem cell transplantation: a retrospective analysis from a tertiary care centre in India
Source: Lancet Reg Health Southeast Asia. 2025 Oct 27;42:100680. doi: 10.1016/j.lansea.2025.100680 (PMC12597296; doi:10.1016/j.lansea.2025.100680)
Supplement: Supplemental Material [file mmc1.pdf]

Supplementary Table -S1: Patients Characteristics

Variable		Total no of Pts N(%)	OS ≤24 months, N=55 (Group I)	OS ≥120 months, N=96 (Group II)	P value
Age (Years)	≤52<br>>52<br><br>≤60<br>>60	221(50.5)<br>217(49.5)<br><br>359(82.0)<br>79(18.0)	22(40.0)<br>33(60.0)<br><br>44(80.0)<br>11(20.0)	52(54.2)<br>44(45.8)<br><br>80(83.3)<br>16(16.7)	0.118
Gender	Male<br>Female	291(66.4)<br>147(33.6)	33(60.0)<br>22(40.0)	65(67.7)<br>31(32.3)	0.193
ISS Stage (n=436)	I	136(31.2)	10(18.2)	39(41.9)	0.001
	II	146(33.5)	13(23.6)	40(41.7)	
	III	154(35.3)	32(58.2)	17(17.7)	
M Protein isotype (n=433)	IgG<br>IgA<br>K+L	258(59.6)<br>72(16.6)<br>103(23.8)	38(70.4)<br>08(14.8)<br>08(14.8)	63(65.6)<br>14(14.6)<br>19(19.8)	0.930
Extramedullary disease, n=437)	Yes<br>No	95(21.7)<br>342(78.3)	24(43.6)<br>31(56.4)	19(19.8)<br>77(80.2)	0.001
Hb (G/dl)	<10<br>≥10	245(55.9)<br>193(44.1)	35(63.6)<br>20(36.4)	49(51.0)<br>47(49.0)	0.091
Platelets ($x10^9$/L N=415	<150<br>≥150	105(25.3)<br>310(74.7)	21(44.7)<br>26(55.3)	12(12.0)<br>81(88.0)	0.001
Albumin (G/dl)	<3.5<br>≥3.5	174(39.7)<br>264(60.3)	33(60.0)<br>22(40.0)	27(28.1)<br>69(71.9)	0.001
eGFR(ml/mt)	<40<br>≥40	100(22.8)<br>338(77.2)	17(30.9)<br>38(69.1)	14(14.6)<br>82(85.4)	0.006
B2M (mcg/L) N=425	<5500<br>≥5500	274(64.5)<br>151(35.5)	22(40.7)<br>32(59.3)	75(81.5)<br>17(16.5)	0.001
Serum LDH N=333	≤300 IU<br>>300 IU	232(69.7)<br>101(30.3)	22(66.7)<br>11(33.3)	43(62.3)<br>26(37.7)	0.356
FISH N=142	Standard<br>High risk	108(83.3)<br>34(16.7)	8(61.5)<br>5(38.5)	16(76.2)<br>05(23.8)	0.353
Serum Calcium N=415	<11.0<br>≥11.0	364(87.7)<br>51(12.3)	44(86.3)<br>07(13.7)	81(89.0)<br>10(11.0)	0.207
BM Plasma cells N=435	≤40%<br>>40%	225(51.7)<br>210(48.3)	24(43.6)<br>31(56.4)	58(60.4)<br>33(39.6)	0.041

Induction regimen	Novel agents<br>VAD<br>Alk agents	350(79.9)<br>68(15.5)<br>20(4.6)	36(65.5)<br>10(18.2)<br>09(16.4)	61(63.5)<br>30(31.3)<br>5(5.2)	0.024
Induction Lines(n=437)	One line<br>>one line	303(69.3)<br>154(30.7)	25(45.5)<br>30(54.5)	71(74.0)<br>25(26.0)	0.002
Melphalan dose (mg/m2)	≤150<br>>150	64(14.6)<br>374(85.4)	10(18.2)<br>45(81.8)	17(17.7)<br>79(82.3)	0.579
Pre-Tx status	Sensitive<br>Resistant	377(86.1)<br>61(13.9)	39(70.9)<br>16(29.1)	85(88.5)<br>11(11.5)	0.020
Interval(months) Diagnosis to Tx	Median (range)<br><br>0-12<br>>12	11.5 (2-90.5)<br><br>246(56.2)<br>192(43.8)	13.5 (4-73)<br><br>24(43.6)<br>31(56.4)	9.0 (4-90.5)<br><br>70(72.9)<br>26((27.1)	 <br>0.001
Transplant in 1st remission	Primary<br>Post salvage	330(75.3)<br>108(26.7)	28(50.9)<br>27(49.1)	86(89.6)<br>10(10.4)	0.001
Post Tx Response, (D+100)	CR<br>Others	301(68.7)<br>137(31.3)	15(37.5)<br>40(72.7)	77(80.2)<br>19(19.8)	0.001
Post Tx Response (day +100)	CR+VGPR<br>others	373(85.2)<br>65(14.8)	28(50.9)<br>27(49.1)	91(94.8)<br>5(5.2)	0.001
Post-transplant response (d+100)	CR+VGPR+PR<br>others	422(96.3)<br>16(3.7)	44(80.0)<br>11(20.0)	96(100.0)<br>0	0.001
CD34+cells (x10(6)/kg	<2<br>≥2	73(17.7)<br>339(82.3)	6(12.8)<br>41(87.2)	30(34.5)<br>57(65.5)	0.005
HCT CI score N=433	0<br>≥1	160(36.7)<br>276(63.3)	23(41.8)<br>32(58.2)	42(44.7)<br>52(55.3)	0.091
Co-morbidities	No<br>Yes	124(28.3)<br>314(71.7)	19(34.5)<br>36(65.5)	29(30.2)<br>67(69.8)	0.428
Diabetes Mellitus	No<br>Yes	368(84.0)<br>70(16.0)	44(80.0)<br>11(20.0)	88(91.7)<br>8(8.3)	0.063
Year of Tx	1995-2005<br>2006-15<br>2016-19	76(17.4)<br>220(50.2)<br>142(32.4)	16(29.1)<br>27(49.1)<br>12(21.8)	32(33.3)<br>64(66.7)<br>0	0.001
Maintenance N=438	Thal<br>Len<br>Borte<br>IFN-a<br>No<br><br>Yes<br>No	148(33.8)<br>162(37.0)<br>51(11.6)<br>28(6.4)<br>49(11.2)<br><br>389(88.8)<br>49(11.2)	12(21.8)<br>13(23.6)<br>02(3.6)<br>02(3.6)<br>26(47.3)<br><br>29(52.7)<br>26(47.3)	53(55.2)<br>09(9.4)<br>11(11.5)<br>17(17.7)<br>06(6.3)<br><br>90(93.8)<br>06(6.3)	 0.001<br><br><br><br><br>0.001

Abbreviations : Thal- thalidomide, Len- lenalidomide, Borte- bortezomib, IFN-a interferon alfa, Tx- transplant, CR-complete response, VGPR- very good partial response

Supplementary table-S2 –

Patients Characteristics : LTS (n=96) versus Others (n=342)

**Variable**		**No of Pts N(%) N=438**	**OS ≥120 months Group II, N=96**	**OS <120 months Group I + III N=342**	**P value**
Age (Years)	≤52<br>>52	221(50.5)<br>217(49.5)	52(54.2)<br>44(45.8)	169(49.4)<br>173(50.6)	0.240
	≤60<br>>60	359(82.0)<br>79(18.0)	80(83.3)<br>16(16.7)	279(81.6)<br>63(18.4)	0.410
Gender	Male<br>Female	291(66.4)<br>147(33.6)	65(67.7)<br>31(32.3)	226(66.1)<br>116(33.9)	0.433
ISS Stage (n=436)	I	136(31.2)	39(41.9)	97(28.5)	0.001
	II	146(33.5)	40(41.7)	106(31.2)	
	III	154(35.3)	17(17.7)	137(40.3)	
M Protein isotype (n=433)	IgG<br>IgA<br>K+L	258(59.6)<br>72(16.6)<br>103(23.8)	63(65.6)<br>14(14.6)<br>19(19.8)	195(57.9)<br>58(17.2)<br>84(24.9)	0.388
Extramedullary disease, n=437)	Yes<br>No	95(21.7)<br>342(78.3)	19(19.8)<br>77(80.2)	76(22.3)<br>265(77.7)	0.355
Hb (G/dl)	<10<br>≥10	245(55.9)<br>193(44.1)	49(51.0)<br>47(49.0)	196(57.3)<br>146(42.7)	0.164
Platelets ($x10^9$/L N=415	<150<br>≥150	105(25.3)<br>310(74.7)	11(12.0)<br>81(88.0)	94(29.1)<br>229(70.9)	0.001
Albumin (G/dl)	<3.5<br>≥3.5	174(39.7)<br>264(60.3)	27(28.1)<br>69(71.9)	147(43.0)<br>195(57.0)	0.005
eGFR(ml/mt)	<40<br>≥40	100(22.8)<br>338(77.2)	14(14.6)<br>82(85.4)	86(25.1)<br>256(74.9)	0.018
B2M (mcg/L) N=424	<5500<br>≥5500	274(64.6)<br>150(35.4)	75(81.5)<br>17(18.5)	199(59.8)<br>134(40.2)	0.001
Serum LDH N=333	≤300 IU<br>>300 IU	232(69.7)<br>101(30.3)	43(62.3)<br>26(37.7)	189(71.6)<br>75(28.4)	0.091
FISH N=142	Standard<br>High risk	108(83.3)<br>34(16.7)	16(76.2)<br>05(23.8)	92(76.0)<br>29(24.0)	0.044
Serum Calcium N=415	<11.0<br>≥11.0	364(87.7)<br>51(12.3)	81(89.0)<br>10(11.0)	283(87.3)<br>41(12.7)	0.412
BM Plasma cells N=435	≤40%<br>>40%	225(51.7)<br>210(48.3)	58(60.4)<br>38(39.6)	167(49.3)<br>172(50.7)	0.034
Induction regimen	Novel agents<br>VAD	350(79.9)<br>68(15.5)	61(63.5)<br>30(31.3)	289(84.5)<br>38(11.1)	0.001

	Alk agents	20(4.6)	5(5.2)	15(4.4)	
Induction Lines(n=437)	One line<br>>one line	303(69.2)<br>135(30.8)	71(74.0)<br>25(26.0)	232(67.8)<br>110(32.2)	0.468
Melphalan dose (mg/m2)	≤150<br>>150	64(14.6)<br>374(85.4)	17(17.7)<br>79(82.3)	47(13.7)<br>295(86.3)	0.207
Pre-Tx status	Sensitive<br>Resistant	377(86.1)<br>61(13.9)	85(88.5)<br>11(11.5)	292(85.4)<br>50(14.6)	0.271
Interval(months) Diagnosis to Tx	Median (range)<br>0-12<br>>12	11.5(2-90.5)<br>246(56.2)<br>192(43.8)	9.0 (4-90.5)<br>70(72.9)<br>26((27.1)	12(2-81)<br>176(51.5)<br>166(48.5)	 0.001
Transplant in 1st remission	Primary<br>Post salvage	330(75.3)<br>108(24.7)	86(89.6)<br>10(10.4)	244(71.3)<br>98(28.7)	0.001
Post Tx Response (D+100)	CR<br>Others	330(75.3)<br>108(31.3)	77(80.2)<br>19(19.8)	224(65.5)<br>118(34.5)	0.004
Post Tx Response (day +100)	CR+VGPR<br>others	373(85.2)<br>65(14.8)	91(94.8)<br>5(5.2)	282(82.5)<br>60(17.5)	0.001
Post-transplant response (day +100)	CR+VGPR+PR<br>others	422(96.3)<br>16(3.7)	96(100.0)<br>0	329(95.4)<br>16(4.6)	0.020
CD34+cells (x10(6)/kg	<2<br>≥2	73(17.7)<br>339(82.3)	30(34.5)<br>57(65.5)	43(13.2)<br>282(86.8)	0.001
HCT CI score N=436	0<br>≥1	160(36.7)<br>276(63.3)	42(44.7)<br>52(55.3)	118(34.5)<br>224(65.5)	0.046
Co-morbidities	No<br>Yes	124(28.3)<br>314(71.7)	29(30.2)<br>67(69.8)	95(27.8)<br>247(72.2)	0.364
Diabetes Mellitus	No<br>Yes	368(84.0)<br>70(16.0)	88(91.7)<br>8(8.3)	280(81.9)<br>62(18.1)	0.012
Year of Tx	1995-05<br>2006-15<br>2016-19	76(17.4)<br>220(50.2)<br>142(32.4)	32(33.3)<br>64(66.7)<br>0	44.0(12.9)<br>156(45.6)<br>142(41.5)	0.001
Maintenance N=437	Thal<br>Len<br>Borte<br>IFN-a<br>None<br><br>Yes<br>No	148(33.8)<br>162(37.0)<br>51(11.6)<br>28(6.4)<br>49(11.2)<br><br>389(88.8)<br>49(11.2)	52(55.9)<br>8(8.6)<br>10(10.8)<br>17(18.3)<br>6(6.0)<br><br>90(93.8)<br>6(6.3)	96(27.8)<br>154(44.6)<br>41(11.9)<br>11(3.2)<br>43(12.5)<br><br>299(87.4)<br>43(12.6)	0.001<br><br><br><br><br><br>0.055

		LTS Group, N=96				Others (Group I +III) N=342			
**Variable**		**Univariate Analysis P value**	**Multivariable analysis**			**Univariate analysis**	**Multivariable analysis**		
			**HR**	**95% CI**	**P value**		**HR**	**95% CI**	**P value**
Age (Years)	≤60 >60	0.027	-	-	-	-	-	-	-
Gender	Male Female	-	-	-	-	0.001	-	-	-
ISS Stage (n=436)	I Vs II Vs III	-	-	-	-	0.033	-	-	-
EM disease, n=437)	Yes No	-	-	-	-	0.001	1.757	1.145-2.695	0.010
Albumin (G/dl)	<3.5 ≥3.5	-	-	-	-	0.017	0.687	0.491-0.961	0.028
B2M (mcg/L) N=423	<5500 ≥5500	-	-	-	-	0.022	-	-	-
Serum LDH N=333U/L	≤300 >300	-	-	-	-	0.032	-	-	-
FISH N=142	Standar High risk	-	-	-	-	0.001	-	-	-
Induction regimen	Novel VAD Alkylat	-	-	-	-	0.001	-	-	-
Induction Lines	One >one	0.06				0.001	-	-	-
Pre-Tx status	Sensitiv Resistan	0.004	0.432	0.191-0.977	0.044	0.001	-	-	-
Interval Diagnosis to Tx(months)	0-12 >12	-	-	-	-	0.081	-	-	--
Transplant in 1st remission	Primary Post salvage	-	-	-	-	0.001	-	-	-
Post Tx, d+100 Response	CR Others	0.035	-	-	-	0.001	0.380	0.239-0.605	0.001
	CR+VG PR others	0.001	0.221	0.066-0.744	0.015	0.001	5.347	2.577-11.095	0.001
Maintenance N=438	Yes No	-	-	-	-	0.001	7.222	4.594-11.353	0.001

Year of Transplant	<2005<br>2006-15<br>2016-19	0.560	-	-	-	0.001	0.619	0.459-0.835	0.002

Supplementary Table 3: Overall Survival – Multivariable Analysis

Variable			LTS Group ,N=96 Multivariable analysis			OTHERS,N=342 Multivariable analysis			
		Univariate	HR	95% CI	P value	Univariate	HR	95% CI	P Value
Age (Years)	≤52<br>>52	0.809	-	-	-	0.555	-	-	-
	≤60<br>>60	0.051	0.482	0.239-0.973	0.042	0.124	-	-	-
Gender	Male<br>Female	0.499	-	-	-	0.183	-	-	-
ISS Stage (n=436)		0.612	-	-	-	0.181	-	-	-
M Protein isotype (n=433)	IgG<br>IgA<br>K+L	0.325	-	-	-	0.970	-	-	-
EM disease, n=437)	Yes<br>No	0.076	2.359	1.223-4.552	0.010	0.091	1.42	0.991-2.039	0.056
Hb (G/dl)	<10<br>≥10	0.679	-	-	-	0.331	-	-	-
Platelets ($x10^9$/L N=415	<150<br>≥150	0.706	-	-	-	0.477	-	-	--
Albumin (G/dl)	<3.5<br>≥3.5	0.288	-	-	-	0.064	1.44	1.079-1.933	0.013
eGFR (ml/mt)	<40<br>≥40	0.909	-	-	-	0.686	-	-	-
B2M (mcg/L) N=423	<5500<br>≥5500	0.530	-	-	-	0.073	0.90	0.671-1.231	0.537
Serum LDH N=333U/L	≤300<br>>300	0.940	-	-	-	0.016	0.88	0.643-1.204	0.425
FISH N=142	Standard<br>High risk	0.362	-	-	-	0.001	0.67	0.468-0.986	0.042
S. Calcium N=415	<11.0<br>≥11.0	0.352	-	-	-	0.797	-	-	-
BM Plasma cells, N=435	≤40%<br>>40%	0.157	-	-	-	0.127	-	-	-
Induction regimen	Novel<br>VAD<br>Alkylat	0.081	0.779	0.284-2.136	0.628	0.001	1.08	0.372-3.143	1.081

Induction Lines (n=437)	One<br>>one	0.124	-	-	-	0.001	1.04	0.666-1.626	0.861
Melphalan dose (mg/m2)	≤150<br>>150	0.698	-	-	-	0.827	-	-	-
Pre-Tx status	Sensitive<br>Resistant	0.004	0.507	0.225-1.142	0.101	0.001	0.51	0.337-0.786	0.002
Interval Diagnosis to Tx(months)	0-12<br>>12	0.302	-	-	-	0.263	-	-	-
Transplant in 1st remission	Primary<br>Post salvage	0.345	-	-	-	0.001	0.67	0.430-1.058	0.086
Post Tx, d+100 Response	CR<br>Others	0.001	0.483	0.234-0.994	0.048	0.001	0.40	0.285-0.584	0.001
	CR+VGPR<br>others	0.001	0.207	0.059-0.725	0.014	0.001	0.80	0.510-1.284	0.368
CD34+cells (x10(6)/kg	<2<br>≥2	0.946	-	-	-	0.317	-	-	-
HCT CI score N=433	0<br>≥1	0.477	-	-	-	0.097	0.67	0.449-0.831	0.002
Co-morbidities	No<br>Yes	0.594	-	-	-	0.894	-	-	-
Diabetes Mellitus	No<br>Yes	0.230	-	-	-	0.379	-	-	-
Maintenance N=438	Yes<br>No	0.792	-	-	-	0.001	0.37	0.244-0.574	0.001

Abbreviations : PFS-progression free survival, mon-months, CR-complete response, VGPR- very good partial response, EM-Extra-medullary, BM- bone marrow, HCT-CI Hematopoietic cell transplantation co-morbidity index

Supplementary Table-4 : Progression Free Survival : Univariate and Multivariable Analysis

	Supplementary Table 5A : Overall Survival							
	**Categorical Variable**					**Continuous variable**		
	Variable	No of Pts	Median OS in months	95% CI	P value	HR	5% CI	P value
Age (Years)	≤52<br>>52	221(50.5)<br>217(49.5)<br><br>359(82.0)<br>79(18.0)	127.0<br>105.0<br><br>107.0<br>118.0	100.6-153.4<br>84.9-125.1<br><br>86.0-128.0<br>102.4-133.6	0.177<br><br><br>0.933	1.005	0.990-1.020	0.520
Hb (G/dl)	<10<br>≥10	245(55.9)<br>193(44.1)	105.0<br>125.0	86.2-123.8<br>93.5-156.5	0.220	0.924	0.878-0.972	0.002
Platelets ($x10^9$/L N=415	<150<br>≥150	105(25.3)<br>310(74.7)	98.5<br>125.0	70.5-126.5<br>103.8-146.2	0.011	1.000	1.0-1.0	0.059
Albumin (G/dl)	<3.5<br>≥3.5	174(39.7)<br>264(60.3)	99.0<br>132.0	73.4-104.6<br>106.8-157.2	0.001	0.806	0.682-0.954	0.012
eGFR (ml/mt)	<40<br>≥40	100(22.8)<br>338(77.2)	103.0<br>119.0	78.4-127.6<br>100.5-137.6	0.152	0.996	0.992-1.000	0.034
B2M (mcg/L) N=423	<5500<br>≥5500	274(64.8)<br>151(35.2)	127.0<br>79.0	107.8-146.2<br>59.1-99.9	0.001	1.0	1.0-1.0	0.657
Serum LDH N=333U/L	≤300<br>>300	232(69.7)<br>101(30.3)	125.0<br>104.0	97.3-152.7<br>80.3-127.7	0.244	1.0	0.999-1.000	0.560
S. Calcium N=415	<11.0<br>≥11.0	364(87.7)<br>51(12.3)	114.5<br>112.0	96.6-132.4<br>95.0-129.0	0.570	1.011	0.926-1.105	0.807
BM Plasma cells, N=435	≤40%<br>>40%	225(51.7)<br>210(48.3)	127.0<br>98.5	96.6-157.4<br>79.9-117.1	0.068	1.007	1.003-1.012	0.002
CD34+cells (x10(6)/kg	<2<br>≥2	422(96.3)<br>16(3.7)	136.6<br>112.0	97.8-174.2<br>98.6-125.4	0.467	1.012	0.949-1.080	0.714
HCT CI score N=436	0<br>≥1	160(17.7)<br>276(63.3)	125.0<br>103.0	95.9-154.1<br>80.4-119.6	0.257	1.115	0.954-1.304	0.171
ISS stage	I<br>II<br>III	136(31.2)<br>146(33.5)<br>154(35.3)	143.0<br>125.0<br>81.0	110.9-175.1<br>90.0-160.0<br>61.2-100.8	0.001			

Myeloma Isotype<br>N=433	IgG<br>IgA<br>Light chain	258(59.6)<br>72(16.6)<br>103(23.8)	112.0<br>115.0<br>125.0	91.0-133.0<br>75.1-154.9<br>93.9-156.1	0.785			
Extramedullary disease, n=437)	Yes<br>No	95(21.7)<br>342(78.3)	72.0<br>115.0	40.6-103.4<br>100.0-130.1	0.004			
FISH<br>N=142	Standard<br>High risk	108(76.1)<br>34(23.9)	130.0<br>97.0	108.1-151.9<br>45.4-148.6	0.039			
Induction regimen	Novel<br>VAD<br>Alk	350(79.9)<br>68(15.5)<br>20(4.6)	119.0<br>97.0<br>32.0	102.0-136.0<br>61.1-132.9<br>12.3-51.7	0.001			
Induction Lines	One line<br>>one line	303(69.2)<br>135(30.8)	130.0<br>60.0	108.6-151.3<br>37.4-82.5	0.001			
Melphalan dose (mg/m2)	≤150<br>>150	64(14.6)<br>374(85.4)	112.0<br>112.0	62.6-161.4<br>97.9-126.1	0.892	1.000	0.994-1.007	0.915
Pre-Tx status	Sensitive<br>Resistant	377(86.1)<br>61(13.9)	125.0<br>57.0	106.2-143.8<br>38.7-75.3	0.001			
Interval (months) Diagnosis to Tx	Median (range)<br><br>0-12<br>>12	11.5<br>(2-90.5)<br><br>246(56.2)<br>192(43.8)	125.0<br>84.0	108.0-142.0<br>59.4-108.6	0.002	1.011	1.002-1.020	0.014
Transplant in 1st remission	Primary<br>Post salvage	330(75.3)<br>108(26.7)	131.0<br>51.0	112.3-149.7<br>38.3-63.7	0.001			
Post Tx, d+100 Response	CR<br>Others	301(68.7)<br>137(31.3)	150.0<br>51.0	119.3-180.7<br>36.8-65.2	0.001			
	CR+VGPR<br>others	373(85.2)<br>65(14.8)	130.0<br>32.0	109.4-150.6<br>19.6-44.4	0.001			
Co-morbidities	No<br>Yes	124(28.3)<br>314(71.7)	121.0<br>109.0	99.7-142.3<br>91.1-126.9	0.831			
Year of Tx	1995-05<br>2006-15<br>2016-19	76(17.4)<br>220(50.2))<br>142(32.4)	85.0<br>104.5<br>-	51.4-118.6<br>86.0-123.0<br>-	0.016			
Maintenance<br>N=438	Yes<br>No	389(88.8)<br>49(11.2)	125.0<br>24.0	106.5-143.5<br>14.4-33.6	0.001			

Abbreviations: Thal- thalidomide, Len- lenalidomide, Borte- bortezomib, IFN-a interferon alfa, Tx- transplant, CR- complete response, VGPR- very good partial response, HCT-CI : hematopoietic cell transplantation comorbidity Index, LTS- Long term survivor

Overall Survival Analysis by Variable Type: Categorical vs. Continuous

Supplementary Table 5B:

Progression free Survival Analysis by Variable Type: Categorical vs. Continuous.

	Categorical Variable					Continuous variable		
		No of Pts	Median PFS in months	95% CI	P value	HR	95% CI	P value
Age (Years)	≤52<br>>52<br><br>≤60<br>>60	221(50.5)<br>217(49.5)<br><br>359(82.0)<br>79(18.0)	61<br>60<br><br>60.0<br>69.5	46.8-75.8<br>47.4-72.6<br><br>48.9-71.1<br>49.6-89.4	0.334<br><br><br>0.864	1.005	0.992-1.018	0.463
Hb (G/dl)	<10<br>≥10	245(55.9)<br>193(44.1)	57.0<br>70.0	45.1-68.9<br>50.3-89.6	0.318	0.949	0.908	0.993
0.022Platelets (x10^9/L N=415	<150<br>≥150	105(25.3)<br>310(74.7)	47.0<br>69.5	28.2-65.8<br>58.6-80.4	0.077	1.0	1.0-1.0	0.164
Albumin (G/dl)	<3.5<br>≥3.5	174(39.7)<br>264(60.3)	50.0<br>74.0	38.9-61.0<br>58.0-90.2	0.003	0.873	0.753-1.012	0.072
eGFR (ml/mt)	<40<br>≥40	100(22.8)<br>338(77.2)	48.0<br>63.5	33.5-62.5<br>51.9-75.1	0.293	0.997	0.994-1.001	0.163
B2M (mcg/L) N=423	<5500<br>≥5500	274(64.8)<br>151(35.2)	70.0<br>48.0	55.4-84.6<br>31.9-64.1	0.009	1.0	1.0-1.0	0.992
Serum LDH N=333U/L	≤300<br>>300	232(69.7)<br>101(30.3)	74.0<br>60.0	57.2-90.8<br>38.9-81.1	0.184	1.0	0.999-1.0	0.519
S. Calcium N=415	<11.0<br>≥11.0	364(87.7)<br>51(12.3)	60.0<br>73.0	50.1-70.0<br>42.2-103.8	0.523	0.960	0.886	1.041
BM Plasma cells, N=435	≤40%<br>>40%	225(51.7)<br>210(48.3)	72.0<br>51.0	56.8-87.2<br>41.1-60.9	0.181	1.005	1.001-1.009	0.018
CD34+cells (x10(6)/kg	<2<br>≥2	73.0<br>339.0	89.5<br>61.0	45.3-133.7<br>52.9-69.1	0.416	1.039	0.977-1.106	0.226
HCT CI score N=436	0<br>≥1	160(17.7)<br>276(63.3)	62.0<br>58.0	46.2-77.8<br>46.7-69.3	0.923	1.038	0.901-1.196	0.607
ISS stage	I<br>II<br>III	136(31.2)<br>146(33.5)<br>154(35.3)	83.0<br>61.0<br>48.0	53.9-112.1<br>46.6-75.4<br>31.4-64.6	0.042			
Myeloma Isotype N=433	IgG<br>IgA<br>Light chain	258(59.6)<br>72(16.6)<br>103(23.8)	66.5<br>53.0<br>57.0	54.8-78.2<br>34.3-71.7<br>27.0-87.0	0.907			

Extramedullary disease, n=437)	Yes<br>No	95(21.7)<br>342(78.3)	34.5<br>66.0	14.5-54.5<br>54.7-77.3	0.025			
FISH N=142	Standard<br>High risk	108(76.1)<br>34(23.9)	88.5<br>69.5	66.5-110.5<br>53.0-86.0	0.104			
Induction regimen	Novel<br>VAD<br>Alk	350(79.9)<br>68(15.5)<br>20(4.6)	70.0<br>33.0<br>20.0	59.6-80.4<br>18.9-47.1<br>11.2-28.8	0.001			
Induction Lines	One<br>>one	303(69.2)<br>135(30.8)	78.5<br>27.0	64.5-92.5<br>18.8-35.2	0.001			
Melphalan dose (mg/m2)	≤150<br>>150	64(14.6)<br>374(85.4)	66.0<br>59.0	30.8-101.2<br>49.0-69.0	0.870	1.001	0.996-1.007	0.702
Pre-Tx status	Sensitive<br>Resistant	377(86.1)<br>61(13.9)	70.0<br>21.0	57.5-82.5<br>15.9-26.1	0.001			
Interval (months) Diagnosis to Tx	Median (range)<br><br>0-12<br>>12	11.5 (2-90.5)<br><br>246(56.2)<br>192(43.8)	70.0<br>50.0	56.6-83.4<br>36.0-64.0	0.008	1.013	1.004-1.022	0.003
Transplant in 1st remission	Primary<br>Post salvage	330(75.3)<br>108(26.7)	76.0<br>24.0	63.1-88.0<br>20.1-27.9	0.001			
Post Tx, d+100 Response	CR<br>Others	301(68.7)<br>137(31.3)	91.0<br>20.0	73.7-108.3<br>17.7-22.3	0.001			
	CR+VGPR<br>others	373(85.2)<br>65(14.8)	75.0<br>16.0	61.8-88.2<br>13.6-18.4	0.001			
Co-morbidities	No<br>Yes	124(28.3)<br>314(71.7)	69.5<br>60.0	45.7-93.3<br>51.0-69.0	0.894			
Year of Tx	1995-05<br>2006-15<br>2016-19	76(17.4)<br>220(50.2))<br>142(32.4)	28.0<br>53.0<br>-	19.5-36.5<br>42.6-63.4<br>-	0.001			
Maintenance N=438	Yes<br>No	389(88.8)<br>49(11.2)	70.0<br>16.0	58.9-81.1<br>11.2-20.8	0.001			

Abbreviations: Thal- thalidomide, Len- lenalidomide, Borte- bortezomib, IFN-a interferon alfa, Tx-transplant, CR- complete response, VGPR- very good partial response, HCT-CI : hematopoietic cell transplantation comorbidity Index, LTS- Long term survivor

Supplementary Table-6: Current Status

Status	No of Pts (%)	LTS Group (n= 96)	Others N=342)	Early Relapse N=55	Intermediate Group N=287
Alive, disease-free	136(31.1%)	42(43.8%)	94(27.5%)	-	94(32.8%)
Alive with disease	31(7.1)	7(7.3%)	24(7.0%)	-	24(8.4%)
Alive in CR2	33(7.5%)	12(12.5%)	21(6.1%)	-	21(6.2%)
Alive with biochemical relapse	1(0.2%)	1(1.0%)	0	-	-
Alive, MGUS like	2(0.5%)	2(2.1%)	0	-	-
Died of Disease	202(46.1%)	25(26.0%)	178(52.0%)	51.0(92.8%)	130(45.3%)
Died of Unrelated cause#	29(6.6)	7(7.3%)	22(6.4%)	3(5.5%)	19(6.6%)
Unknown	01(0.2%)	-	1(0.3%)	1(1.8%)	-
Total	438	96	342	55.0	287

P value LTS versus Others ,p <0.001, #-this includes 11 patients who had second primary malignancy, In 6 of these , myeloma was in remission at the time of death.

Supplementary Table-7 : Review of Literature

1st Author(Ref)	Period of study/ No of Pts	Criteria % long term	Factors	Post Tx maintenance
Pasvolsky,2024 MDACC, USA (8)	2000-2014 N=1576, NDMM	PFS≥8 Yr 255(15%)	Younger age HRCA=4% Lower RISS Lower BMPC%	63%
Oliver Caldes 2022, Spain(9)	1990-2015 N=250 NDMM	Sustained response for ≥5 yr 54(21.6%) long term responders	Younger age, Female gender, Better ECOG PS, Lower ISS stage Lower BM PC% HRCA=9%,Low CRP and LDH,Lower (s) Cal.	57.4%
Nishimura et al 2020 (10)	1989-2018 N=4329	PFS Cure fraction improved over time, 17.3 % - 20%	Year of transplant Female gender Age <65 yr Participation in total therapy protocols	total therapy protocols
Paquin , 2020 Mayo Clinic Rochester,USA (11)	1998-2006 N=509 Tx within 12 months	PFS>8 Yrs 46(9%)	Median age 57 Yr 61% females ISSI=54% HRCA=2.5%	No maintenance
Terpos , 2020 Greece(12)	1994-2010 N=406	PFS≥7 Yr 36(8.8%)	Younger age Better ECOG PS Higher Hb Better creatinine clearance Lower ISS HRCA=0%	50%
Lehners,2018 Germany (7)	1993-2014 N=865	Achievement of CR post Tx	Old age Low platelet counts ISS III	44.6%
Usmani et al IMWG study 2018(6)	1990-2006 N=7291 International study Age up to 75 Yr, Tx: 52% Median FU: 5.8 Yr	10 year OS 14.37%, cure fraction	Negative association with 10 Yr OS : Age >65 Y, IgA isotype Low albumin, Elevated B2M,(s) creatinine ≥2 mg%, Hb<10 G%, Platelets <150x10(9)/L	Not mentioned
Present Study	N=438 1995-2019	≥10 Year OS N=96(21.9%)	Pre-Tx chemo-sensitive disease post- Tx CR+VGPR	88.8%

Abbreviations : NDMM- newly diagnosed multiple myeloma, Tx- transplant, Y-year, PFS- progression free survival, HRCA- high risk cytogenetic abnormality, ISS- International staging

system, RISS- revised ISS, ECOG : PS Eastern Cooperative Oncology Group performance status, BMPC- bone marrow plasma cell ,

Supplementary Table 8: Year Wise – Key Outcomes

	Factors	**≤2005**	**2006-2015**	**2016-19**	P value
No of Patients		76(17.4%)	220(50.2%)	142(32.4%)	
Age (Y)	Median (range)	53.0 (29-67)	53.0 (29-68)	52.0 (20-72)	0.096
Induction	Novel agents<br>VAD<br>Alkylating agents	3(3.9)<br>55(72.4)<br>18(23.7)	205(93.2)<br>13(5.9)<br>02(0.9)	142(100.0)<br>-<br>-	0.001
Pre Tx status	Sensitive<br>Resistant	46(60.5)<br>30(39.5)	201(91.4)<br>19(8.6)	130(91.5)<br>12(8.5)	0.001
Post Transplant response	CR(%)	35(46.1)	152(69.1)	114(80.3)	0.001
	CR+VGPR(%)	53(69.7)	188(85.5)	132(93.0)	0.001
	CR+VGPR+PR(%)	69(90.8)	212(96.4)	141(99.3)	0.006
	<PR(%)	7(9.2)	8(3.6)	01(0.7)	
Post transplant maintenance	Thalidomide<br>Lenalidomide<br>Bortezomib<br>IFN-alfa<br>No	35(46.1)<br>0<br>0<br>28(36.8)<br>13(17.1)	104(47.3)<br>43(19.5)<br>41(18.6)<br>0<br>32(14.5)	9(6.3)<br>119(83.8)<br>10((7.0)<br>0<br>4(2.8)	0.001
PFS (Months)	Median (95% CI)	28.0 19.5-36.5)	53.0 (42.8-63.2)	NR Mean=72.9 (69.2-78.5)	0.001
OS (Months)	Median (95% CI)	85.0 (51.4-118.6)	104.5 (86.0-123.0)	NR Mean 83.9 (78.9-88.8)	0.010
Current Status	Alive, n=207	14(6.8)	91(41.4)	102(71.8)	0.001
	Died of disease, n=202	50(24.8)	120(54.5)	32(22.5)	0.001
	Died of unrelated causes, N=29	12(15.8)	9(4.1)	8(5.63)	0.001
